# Mannose-binding lectin deficiency is associated with early onset of polyarticular juvenile rheumatoid arthritis: a cohort study

**DOI:** 10.1186/ar2386

**Published:** 2008-03-11

**Authors:** Koert M Dolman, Nannette Brouwer, Florine NJ Frakking, Berit Flatø, Paul P Tak, Taco W Kuijpers, Øystein Førre, Anna Smerdel-Ramoya

**Affiliations:** 1 Department of Pediatric Hematology, Immunology and Infectious diseases, Emma Children's Hospital, Academic Medical Center, University of Amsterdam, Meibergdreef, Amsterdam, 1105 AZ, The Netherlands; 2 Department of Blood Cell Research, Sanquin Research at CLB, and Landsteiner Laboratory, University of Amsterdam, Plesmanlaan, Amsterdam, 1066 CX, The Netherlands; 3 Department of Rheumatology, Rikshospitalet University Hospital, Sognsvannsveien, Oslo, NO-0027, Norway; 4 Division of Clinical Immunology and Rheumatology, Academic Medical Center, University of Amsterdam, Meibergdreef, Amsterdam, 1105 AZ, The Netherlands

## Abstract

**Background:**

Mannose-binding lectin (MBL) is an innate immune protein. The aim of our study was to determine whether genetically determined MBL deficiency is associated with susceptibility to juvenile rheumatoid arthritis (JRA) and whether *MBL2 *genotypes are associated with JRA severity.

**Methods:**

In a retrospective cohort study of 218 patients with polyarthritis (n = 67) and oligoarthritis (n = 151), clinical and laboratory disease variables were obtained by clinical examination and chart reviews. Healthy Caucasian adults (n = 194) served as control individuals. *MBL2 *gene mutations were determined by Taqman analysis to identify genotypes with high, medium and low expression of MBL. Functional MBL plasma concentrations were measured using enzyme-linked immunosorbent assay. Associations between clinical and laboratory variables and *MBL2 *genotypes were determined by Kruskal-Wallis and χ^2 ^tests.

**Results:**

*MBL2 *genotype frequencies were similar in polyarthritis and oligoarthritis patients as compared with control individuals. MBL plasma concentrations were associated with the high, medium and low MBL genotype expression groups (*P *< 0.01). In polyarthritis patients, the presence of low-expressing (deficient) *MBL2 *genotypes was associated with early age at onset of disease (*P *= 0.03). In oligoarthritis patients, patients with low-expressing *MBL2 *genotypes were more often in remission (81%) than patients in the medium (54%) and high (56%) genotype groups (*P *= 0.02). The remaining clinical and laboratory variables, such as arthritis severity index, presence of radiographic erosions and antinuclear antibody positivity, were not associated with *MBL2 *genotypes.

**Conclusion:**

Genetically determined MBL deficiency does not increase susceptibility to JRA, but MBL deficiency is associated with a younger age at onset of juvenile polyarthritis. On the other hand, MBL-deficient children with juvenile oligoarthritis are more often in remission. Therefore, MBL appears to play a dual role in JRA.

## Introduction

Juvenile rheumatoid arthritis (JRA), also known as juvenile idiopathic arthritis (JIA), is a rheumatic disease of childhood, and includes a heterogeneous group of patients with differing characteristics, clinical manifestations, serological parameters and genetic background. Although the aetiology of JRA remains unknown, it appears to be a combined action of environmental, hormonal and genetic factors [1-3]. It is generally believed that infections play an important role in the pathogenesis of JRA [4].

Mannose-binding lectin (MBL) is a serum protein, produced in the liver, that plays an important role in innate immunity and functions as an opsonin, recognizing sugar structures on a wide variety of micro-organisms [5]. Serum MBL can directly opsonize micro-organisms and enhance the uptake by phagocytic cells via activation of the lectin pathway of the complement system [6,7]. Genetically determined functional MBL serum levels vary within the population. Six single nucleotide polymorphisms (SNPs) in the *MBL2 *gene on chromosome 10 are known to influence MBL plasma levels. Reduced or deficient MBL plasma levels are seen in individuals with heterozygous or homozygous SNPs in codons 54 (*B *mutation), 52 (*D *mutation), or 57 (*C *mutation) of exon 1 of the *MBL2 *gene [5,8,9]. The variant alleles occur with a combined phenotype frequency of about 25% to 30% in the Caucasian population [10,11]. The wild-type is called *A*, whereas the common designation for the variant alleles is *O*. In addition, MBL plasma concentrations fluctuate in the presence or absence of three SNPs (position -550: *H *and *L *alleles; position -221: *X *and *Y *alleles; and position +4: *P *and *Q *alleles) in the promoter region of the *MBL2 *gene [12,13]. However, only the *X*/*Y *variant has a pronounced influence; the *X *allele is associated with decreased plasma MBL levels and the *Y *variant with high plasma MBL levels. Subsequently, intermediately decreased MBL serum levels are seen in individuals with the genotypes *XA*/*XA *and *YA*/*O*, whereas very low or undetectable serum MBL levels are seen in individuals with genotypes *XA*/*O *and *O*/*O*. Individuals with *YA*/*YA *and *YA*/*XA *haplotypes have high or normal MBL levels. Therefore, patients can be classified into high (*YA*/*YA *and *YA*/*XA*), medium (*XA*/*XA *and *YA*/*O*) and low (*XA*/*O *and *O*/*O*) MBL genotype expression groups [10,14].

MBL deficiency has been associated with increased susceptibility to and severity of infections, especially in children [15,16]. In addition, it has been suggested that MBL modulates inflammation and autoimmune disease; for example, variant MBL alleles are risk factors for systemic lupus erythematosus [17,18]. It has also been suggested that MBL deficiency is associated with joint erosions and early disease onset of adult rheumatoid arthritis (RA) [19-23], although other investigators were unable to confirm such an association [24,25]. Moreover, it is believed that MBL plays an important role in innate immunity. Although unproven, it has been hypothesized that infection may trigger JRA in genetically susceptible patients [26]; this viewpoint suggests that MBL deficiency can predispose to JRA. In a recently reported study [27], there was no significant difference in genotypic frequencies of *MBL2 *codon 54 SNPs between 93 patients with JIA and 48 healthy control individuals. Codon 57 SNPs were not found. The other *MBL2 *SNPs were not investigated in this study. In addition, no association of *MBL2 *haplotypes was found between the subgroups of patients with JIA and control individuals.

The aim of the present study was to determine whether genetically determined MBL deficiency is associated with susceptibility to JRA and whether *MBL2 *genotypes are associated with severity of JRA, as assessed based on patient characteristics and disease variables.

## Materials and methods

### Patients and samples

Eligible patients participated in a larger cohort study of Caucasian Norwegian children with JRA and visited the Department of Rheumatology of Rikshospitalet University Hospital (Oslo, Norway) for the first time between January 1980 and September 1985 [28,29]. JRA was defined as meeting the American College of Rheumatology criteria for JRA [30]. The 236 patients from whom blood was drawn were stratified according to JRA subgroup, because disease variables vary within these groups. Patients with systemic arthritis (n = 2) and juvenile spondylarthropathy (juvenile ankylosing spondylitis [n = 3], seronegative enthesopathy [n = 4], juvenile psoriatic arthritis [n = 11], or inflammatory bowel disease associated arthritis [n = 1]) were excluded because these subgroups consisted of too few individuals to permit reliable statistic analysis. Of the 218 remaining patients, 151 had oligoarthritis and 67 had polyarthritis. The patients were examined and interviewed after a median disease duration of 14.8 years (interquartile range [IQR] 13.5 to 16.2 years) and their medical records were reviewed for variables associated with the onset and course of disease.

Plasma samples were immediately frozen at -80°C. Genomic DNA was isolated from heparinized/EDTA blood according to standard procedures. The study is compliant with the Helsinki Declaration. It was approved by the Regional Ethics Committee for Medical Research and written informed consent was given by the parents. Routine laboratory investigations included C-reactive protein (CRP) level and erythrocyte sedimentation rate, and detection of IgM-rheumatoid factor (RF) and antinuclear antibodies (ANAs). In addition, MBL plasma concentrations and genotypes were determined in 194 healthy adult volunteers, who served as control individuals [10].

### Clinical data

Demographic and clinical outcome variables were recorded from the charts at the follow-up visit. Onset of disease was defined as the date that arthritis was documented by a physician for the first time. The clinical examination included a physician's global assessment (PGA) of overall disease activity (ranging from 0 to 5) as well as assessment of numbers of actively involved (swollen or tender and mobility-restricted) and affected (swollen or mobility-restricted) joints, disease remission status (current remission, active disease after previous remission, or continuously active disease) and presence of uveitis. Furthermore, the number of cumulative affected joints and the arthritis severity index score were recorded. The Childhood Health Assessment Questionnaire (CHAQ) was used to measure physical disability at follow up [31]. It measures physical functioning in the following areas: dressing and grooming, arising, eating, walking, hygiene, reaching, gripping and activities. The mean CHAQ score ranges from 0 to 3, where 0 represents no disability and values above 1.5 represent severe disability.

### Radiographic examinations

Radiographs of the sacroiliac joints, hips, ankles and tarsi were obtained at follow up of all patients, and examined by two radiologists, who were blinded to patient information and had no access to earlier radiographic, clinical, or laboratory data. Radiographs of other affected joints were obtained when clinically indicated. The radiographic changes were classified as joint erosions (grades III to V) or no joint erosions (grades 0 to II).

### MBL assays

MBL measurements were performed at Sanquin Research and the Landsteiner Laboratory (Academic Medical Center, Amsterdam, The Netherlands). MBL plasma levels were measured using an enzyme-linked immunosorbent assay, as previously described [14,32]. Briefly, mannose was coated to the solid phase, and after incubation with plasma, biotinylated mouse-anti-human MBL IgG (10 μg/ml; Tacx and coworkers [32], Amsterdam) was used as detection antibody [32].

Genotyping of the promoter polymorphisms and exon 1 SNPs was performed by allelic discrimination using a Taqman assay, using specific primers and minor groove binding probes for each SNP [14,33]. Genotyping was performed independently of the clinical data collection and MBL plasma level measurements. Patients were classified into three *MBL2 *genotype groups with high, medium and low expression of MBL. The influence of the *X*/*Y *allele was also determined by studying six 'extended' genotype groups: *YA*/*YA*, *YA*/*XA*, *XA*/*XA*, *YA*/*O*, *XA*/*O *and *O*/*O*.

### Statistical analysis

Data are presented as median and IQR because clinical and laboratory variables were not normally distributed. Consequently, the nonparametric Kruskal-Wallis and Mann-Whitney U tests were used for comparison of these variables. Frequencies between groups were compared by the χ^2 ^or Fisher's exact test, where appropriate. Multivariate binominal logistic regression was used to study the association between *MBL2 *genotype and remission status (active/remission) after adjustment for disease duration. The odds ratio and 95% confidence interval were calculated. *P *< 0.05 was considered statistically significant. Patients were stratified according to remission status (active/remission) to explore further the association between CRP levels and *MBL2 *genotype in oligoarthritis patients. For statistical analysis SPSS 12.0.1 software was used (SPSS Inc., Chicago, IL, USA).

## Results

### Demographics

The patient group consisted of 59 boys (27%) and 159 girls (73%), with a median age at diagnosis of 8.0 years (range 0.8 to 15.4 years; Table 1). The median (IQR) follow-up time was 14.8 (13.6 to 16.2) years. Table 1 shows that most patient characteristics differ between polyarthritis and oligoarthritis patients (*P *< 0.05). Therefore, the association between *MBL2 *genotype and disease was analyzed in the two JRA subsets separately (see below).

**Table 1:** Demographic, clinical, and laboratory characteristics of JRA patients, according to disease onset subtype

Characteristic	All JRA patients (n = 218)	JRA subgroups		*P*
		Polyarthritis (n = 67)	Oligoarthritis (n = 151)	
Demographic variables				
Males (*n *[%])	59 (27%)	19 (28%)	40 (27%)	0.87
Age (years) at onset	8.0 (3.7 to 11.6)	9.4 (5.5 to 12.9)	7.3 (3.1 to 11.5)	<0.01
Disease duration (years) at follow up	14.8 (13.6 to 16.2)	14.6 (13.4 to 16.3)	15.0 (13.8 to 16.2)	0.68
Clinical variables				
Number of cumulative affected joints	5 (2 to 15)	20 (11 to 34)	4 (2 to 6)	<0.01
Arthritis severity index	2 (0 to 11)	12 (2 to 37)	2 (0 to 5)	<0.01
Physician global assessment	1 (1 to 2)	2 (1 to 3)	1 (1 to 2)	<0.01
Childhood Health Assessment Questionnaire score	0 (0 to 0.4)	0.1 (0 to 0.6)	0 (0 to 0.3)	<0.01
Patients with uveitis (*n *[%])	42 (19%)	10 (15%)	34 (23%)	0.27
Remission status at follow-up (*n *[%])				
Current remission	122 (56%)	32 (48%)	90 (60%)	<0.01
Active but previous remission	55 (25%)	14 (21%)	41 (27%)	
Continuously active	41 (19%)	21 (31%)	20 (13%)	
Radiographic erosions grade III to IV (*n *[%])	51 (23%)	30 (45%)	21 (14%)	<0.01
Laboratory variables				
Erythrocyte sedimentation rate (mm/hour)	6 (4 to 13)	7 (4 to 22)	6 (4 to 11)	0.19
C-reactive protein (mg/l)	5 (3 to 6)	5 (3 to 14)	5 (1 to 5)	<0.01
Antinuclear antibody positivity	79 (36%)	17 (26%)	62 (41%)	0.03
IgM-rheumatoid factor positivity	11 (5%)	11 (16%)	0 (0%)	<0.01

Continuous variables are presented as median (interquartile range [IQR]). JRA, juvenile rheumatoid arthritis.

### MBL genotype and functional MBL levels in relationship to disease

**Figure 1:**
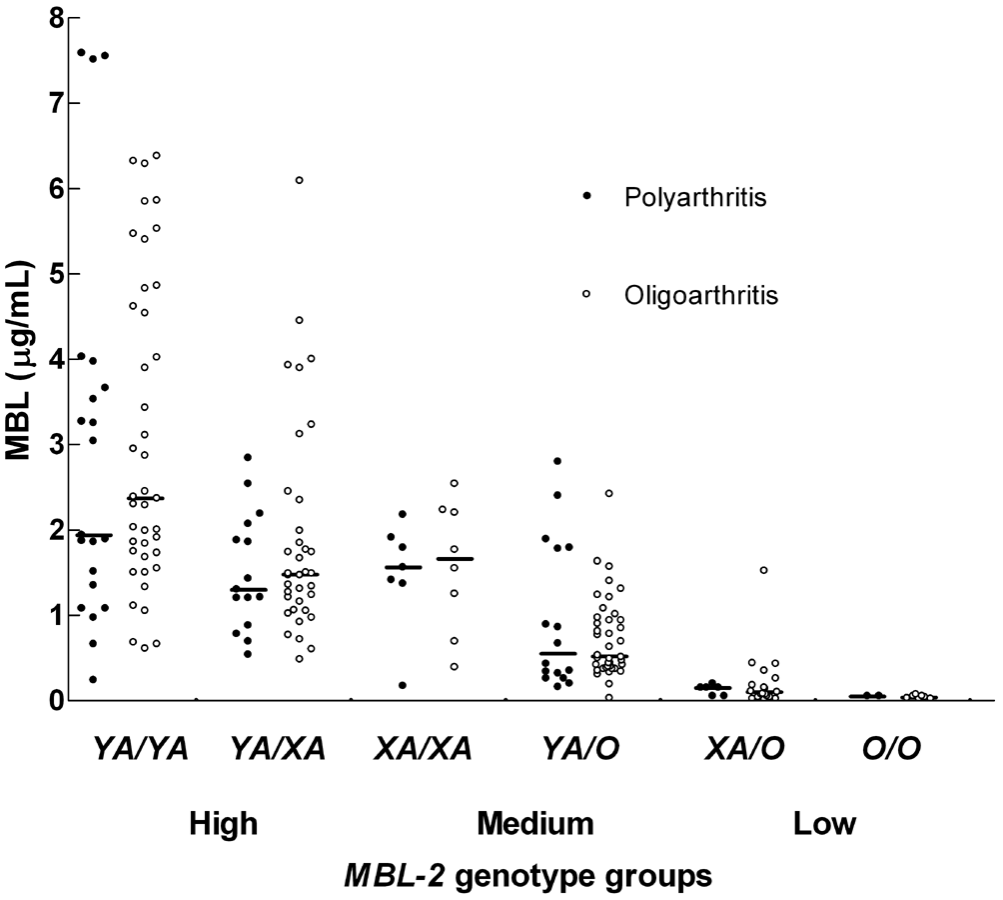
MBL level according to (extended) *MBL2 *haplotypes in patients with juvenile polyarthritis and oligoarthritis. Median mannose-binding lectin (MBL) plasma levels, represented by horizontal lines, differ between extended haplotype groups (*P *< 0.01), but not between patients with oligoarthritis (n = 151) and polyarthritis (n = 67) who had similar haplotypes (*P *> 0.46).

The median (range) MBL plasma concentration was 1.23 (0.01 to 7.59) μg/ml in the 218 JRA patients. Frequencies of the *B*, *C *and *D *exon 1 mutations in these JRA patients did not differ significantly from those in control individuals (*P *= 0.89, *P *= 1.00 and *P *= 0.37, respectively; Table 2). No deviation from Hardy-Weinberg equilibrium was observed in JRA patients or healthy control individuals (data not shown). Of the 218 JRA patients, 113 (52%) were in the high genotype expression group, 71 (33%) were in the medium genotype group and 34 (16%) were in the low genotype expression group (Table 2). The frequency of MBL deficiency was similar in JRA patients and control individuals (odds ratio 1.1, 95% confidence interval 0.9 to 1.4; *P *= 0.37). The distribution of the extended *MBL2 *haplotypes in the 218 JRA patients was as follows: 62 (28%) *YA*/*YA *haplotype, 51 (23%) *YA*/*XA *haplotype, 15 (7%) *XA*/*XA *haplotype, 56 (26%) *YA*/*O *haplotype, 25 (12%) *XA*/*O *haplotype and 9 (4%) *O*/*O *haplotype. These frequencies did not differ significantly from those in control individuals (*P *= 0.89) or between the two JRA subgroups (*P *= 0.69). MBL plasma concentrations were highest in the *YA*/*YA *genotype group and almost absent in *XA*/*O *and *O*/*O *groups (Figure 1). In JRA patients with high, medium and low expressing haplotypes, the median (IQR) MBL plasma level was 1.86 (1.23 to 3.26) μg/ml, 0.77 (0.38 to 1.41) μg/ml and 0.07 (0.04 to 0.15) μg/ml, respectively (*P *< 0.01; Table 2). The MBL plasma concentrations of the six extended genotype groups did not differ between polyarthritis and oligoarthritis patients (*P *> 0.46).

**Table 2:** MBL concentrations and *MBL2 *genotypes

	Control individuals	All JRA patients	JRA subgroups	
			Polyarthritis	Oligoarthritis
Exon 1 mutations				
Sum *A*/*A*	120 (62)	128 (59)	43(64)	85 (56)
Sum *A*/*O*	65 (33)	81 (37)	22 (33)	59 (39)
*A*/*B*	40 (21)	43 (20)	14 (21)	29 (19)
*A*/*C*	5 (3)	7 (3)	0 (0)	7 (5)
*A*/*D*	20 (10)	31 (14)	8 (12)	23 (15)
Sum *O*/*O*	9 (5)	9 (4)	2 (2)	7 (5)
*B*/*B*	4 (2)	4 (2)	1 (1)	3 (2)
*B*/*C*	1 (0)	0 (0)	0 (0)	0 (0)
*B*/*D*	2 (1)	3 (1)	1 (1)	2 (1)
*C*/*D*	0 (0)	1 (0)	0 (0)	1 (1)
*D*/*D*	2 (1)	1 (0)	0 (0)	1 (1)
Total	194 (100)	218 (100)	67 (100)	151 (100)
Genotype groups				
High	110 (57)	113 (52)	36 (54)	77 (51)
*YA*/*YA*	60 (31)	62 (28)	21 (31)	41 (27)
*YA*/*XA*	50 (26)	51 (23)	15 (22)	36 (24)
Medium	52 (27)	71 (33)	23 (34)	48 (32)
*XA*/*XA*	10 (5)	15 (7)	7 (10)	8 (5)
*YA*/*O*	42 (22)	56 (26)	16 (24)	40 (27)
Low	32 (16)	34 (16)	8 (12)	26 (17)
*XA*/*O*	23 (12)	25 (12)	6 (9)	19 (13)
*O*/*O*	9 (4)	9 (4)	2 (3)	7 (5)
Total	194 (100)	218 (100)	67 (100)	151 (100)
MBL concentration				
High	1.65 (1.20 to 2.69)	1.86 (1.23 to 3.26)	1.87 (1.14 to 3.15)	1.85 (1.32 to 3.67)
Medium	0.52 (0.40 to 0.92)	0.77 (0.38 to 1.41)	0.89 (0.32 to 1.79)	0.73 (0.38 to 1.43)
Low	0.04 (0.02 to 0.13)	0.07 (0.04 to 0.15)	0.10 (0.05 to 0.15)	0.07 (0.04 to 0.17)

Norwegian Caucasian children with juvenile polyarthritis (n = 67) and oligoarthritis (n = 151) are compared with 194 healthy Dutch Caucasian adult control individuals. Values are expressed as number (%) or, for continuous variables, as median (interquartile range). Median mannose-binding lectin (MBL) concentrations and frequencies of exon 1 mutations and *MBL2 *genotype groups did not differ between all juvenile rheumatoid arthritis (JRA) patients and healthy control individuals or within the polyarthritis and oligoarthritis groups (*P *values > 0.05). *A *is the designation for wild-type; *O *is the common designation for the variant alleles *B *(codon 54), *C *(codon 57) and *D *(codon 52).

### MBL association with disease parameters

#### Polyarthritis group

**Figure 2:**
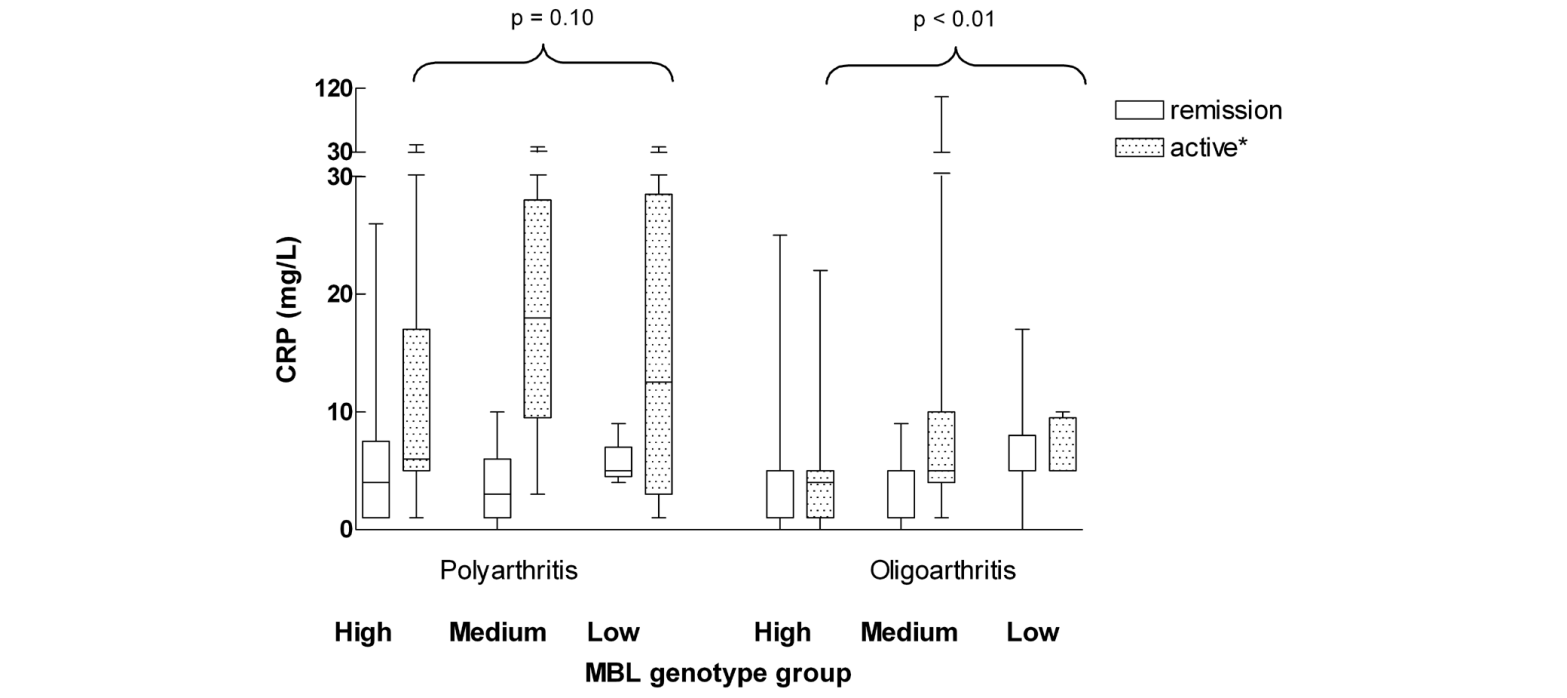
CRP and *MBL2 *genotype: remission versus active disease. Shown are serum C-reactive protein (CRP) concentrations (mg/l) and mannose-binding lectin (MBL) genotype in patients with a current remission versus active disease (either active disease with a previous remission or continuously active disease). *Only CRP values of oligoarthritis patients with active disease (as compared with patients with a current remission) differed statistically significantly (*P *< 0.01).

In the 67 patients with polyarthritis, patients in the low *MBL2 *genotype group were younger (4.4 years, IQR 3.6 to 7.0 years) at onset of disease than the patients in the medium (10.1 years, IQR 8.4 to 13.0 years) and high (9.5, IQR 5.6 to 13.0 years) genotype groups (*P *= 0.05; Table 3). This association was even stronger after exclusion of the 11 IgM-RF positive patients (*P *= 0.02; data not shown). The same association was found in the ANA-negative (*P *< 0.01) but not in the ANA-positive patients (*P *= 0.47; data not shown). In the high genotype expression group, four patients (11%) were IgM-RF positive, as compared with seven patients (30%) in the medium genotype group and none in the low genotype group (*P *= 0.06). We did not find any association of MBL genotype groups with other clinical features, such as number of cumulative affected joints, arthritis severity index, PGA, CHAQ scores, or number of patients with uveitis, remission, or severe radiographic erosions, or with laboratory tests such as ANAs, erythrocyte sedimentation rate, and IgM-RF (Table 3). CRP levels were similar in the high, medium and low MBL2 genotype group (Table 3), even after stratification for remission status (*P *> 0.10; Figure 2). No differences in clinical or laboratory variables were found between patients with the *A*/*A*, the *A*/*O *and the *O*/*O MBL2 *genotypes either (data not shown).

**Table 3:** Association of demographic, clinical, and laboratory characteristics and *MBL2 *genotype expression groups: juvenile polyarthritis

Characteristic	*MBL *genotype expression groups			*P*^a^	*P*^b^
	High (n = 36)	Medium (n = 23)	Low (n = 8)		
Demographic variables					
Males	10 (28%)	4 (17%)	5 (63%)	0.05	0.04
Age (years) at onset	9.5 (5.6 to 13.0)	10.1 (8.4 to 13.0)	4.4 (3.6 to 7.0)	0.03	<0.01
Disease duration (years) at follow up	14.6 (13.5 to 16.3)	14.5 (13.2 to 16.4)	15.8 (13.4 to 16.6)	NS	NS
Clinical variables				NS	NS
Cumulative affected joints	18 (10 to 32)	22 (10 to 36)	23 (12 to 39)	NS	NS
Actively involved joints	1 (0 to 4)	2 (0 to 8)	0 (0 to 2)	NS	NS
Affected joints	6 (2 to 18)	8 (0 to 20)	8 (1 to 27)	NS	NS
Arthritis severity index	10 (2 to 31)	17 (0 to 46)	19 (2 to 54)	NS	NS
Physician global assessment	2 (1 to 3)	2 (1 to 4)	1 (1 to 2)	NS	NS
Childhood Health Assessment Questionnaire score	0.1 (0.0 to 0.6)	0.3 (0.0 to 1.2)	0 (0.0 to 0.3)	NS	NS
Patients with uveitis	5 (14%)	4 (17%)	1 (13%)	NS	NS
Remission status at follow up				NS	NS
Current remission	18 (50%)	10 (44%)	4 (50%)		
Active, but previous remission	8 (22%)	4 (17%)	2 (25%)		
Continuously active	10 (28%)	9 (39%)	2 (25%)		
Radiographic erosions grade III to IV	16 (44%)	10 (44%)	4 (50%)	NS	NS
Laboratory variables				NS	NS
Erythrocyte sedimentation rate (mm/hour)	8 (4 to 20)	8 (5 to 25)	3 (0 to 23)	NS	NS
C-reactive protein (mg/l)	5 (3 to 9)	7 (3 to 18)	5 (4 to 17)	NS	NS
Antinuclear antibody positivity	10 (28%)	6 (27%)	1 (13%)	NS	NS
IgM-rheumatoid factor positivity	4 (11%)	7 (30%)	0 (0%)	NS	NS

Included in this analysis are 67 patients with juvenile polyarthritis. Values are expressed as number (%) or, for continuous variables, as median (interquartile range). ^a^Comparison of the high, medium and low genotype expression groups by means of the two-sided Fisher's exact test and Kruskal-Wallis test. ^b^Comparison of the high and medium genotype group versus the low genotype expression group by means of the two-sided Fisher's exact test and Mann-Whitney U-test. NS, not significant.

#### Oligoarthritis group

In the 151 oligoarthritis patients, age at onset was similar in the high, medium and low genotype expression groups (*P *= 0.66; Table 4). Patients with oligoarthritis carrying the low MBL expression genotype were more often in remission (81%) than patients in the medium (54%) and high (56%) genotype groups (*P *= 0.02; Table 4). Multivariate analysis revealed that, after adjustment for disease duration, patients in the low genotype groups had an odds ratio of 2.5 (95% confidence interval 1.1 to 5.7) of being in remission at follow up, as compared with patients in the high genotype group (*P *= 0.04; data not shown). The median CRP level was 5 mg/l at follow up in the three genotype groups, but the CRP value distribution differed statistically significantly (*P *< 0.01; Table 4) between these three groups. Figure 2 shows CRP levels and *MBL2 *genotypes in patients with a current remission and patients with active disease with or without a previous remission. When the patients were stratified according to remission status (remission versus active), median CRP levels remained statistically significantly different in patients with active disease as compared with those with a current remission. In these patients, the median (IQR) CRP level was 4 (1 to 5) mg/l in the high genotype group versus 5 (4 to 10) mg/l in the medium and 5 (5 to 9) mg/l in the low genotype groups (*P *< 0.01).

**Table 4:** Association of demographic, clinical, and laboratory characteristics and *MBL2 *genotype expression groups: oligoarthritis

Characteristic	*MBL *genotype expression groups			*P*^a^	*P*^b^
	High (n = 77)	Medium (n = 48)	Low (n = 26)		
Demographic variables					
Males	22 (29%)	10 (21%)	8 (31%)	NS	NS
Age (years) at onset	6.6 (3.4 to 10.6)	8.7 (2.6 to 12.1)	6.6 (2.9 to 12.6)	NS	NS
Disease duration (years) at follow up	14.5 (13.6 to 15.9)	15.1 (13.2 to 16.1)	15.3 (14.1 to 16.4)	NS	NS
Clinical variables				NS	NS
Cumulative affected joints	3 (2 to 6)	4 (2 to 6)	2 (2 to 4)	NS	NS
Actively involved joints	0 (0 to 1)	0 (0 to 1)	0 (0 to 0)	NS	NS
Affected joints	1 (0 to 3)	1 (0 to 2)	1 (0 to 3)	NS	NS
Arthritis severity index	2 (0 to 6)	2 (0 to 5)	1.5 (0 to 5)	NS	NS
Physician global assessment	1 (1 to 2)	1 (1 to 2)	1 (1 to 2)	NS	NS
Childhood Health Assessment Questionnaire score	0.0 (0.0 to 0.3)	0.0 (0.0 to 0.1)	0.0 (0.0 to 0.4)	NS	NS
Patients with uveitis	12 (16%)	16 (33%)	6 (23%)	NS	NS
Remission status at follow up				0.02	0.01
Current remission	43 (56%)	26 (54%)	21 (81%)		
Active, but previous remission	27 (35%)	13 (27%)	1 (4%)		
Continuously active	7 (9%)	9 (19%)	4 (15%)		
Radiographic erosions grade III to IV	11 (14%)	8 (17%)	2 (8%)	NS	NS
Laboratory variables				NS	NS
Erythrocyte sedimentation rate (mm/hour)	6 (4 to 11)	8 (5 to 13)	5 (4 to 11)	NS	NS
C-reactive protein (mg/l)	5 (1 to 5)	5 (3 to 6)	5 (5 to 9)	<0.01	0.01
Antinuclear antibody positivity	33 (43%)	18 (38%)	11 (42%)	NS	NS
IgM-rheumatoid factor positivity	0	0	0	-	-

Included in this analysis are 151 patients with juvenile oligoarthritis. Values are expressed as number (%) or, for continuous variables, as median (interquartile range). ^a^Comparison of the high, medium and low genotype expression groups by means of the two-sided Fisher's exact test and Kruskal-Wallis test. ^b^Comparison of the high and medium genotype group versus the low genotype expression group by means of the two-sided Fisher's exact test and Mann-Whitney U-test. NS, not significant.

The remaining clinical and laboratory variables did not differ between the patients in the high, medium and low *MBL2 *genotype groups (Table 4). The differences found in CRP level and remission status were also present in patients with the *A*/*A*, the *A*/*O *and the *O*/*O MBL2 *genotype. Other clinical and laboratory variables did not differ between these patients (data not shown).

## Discussion

In this study we demonstrated that the frequency of MBL deficiency was not increased in 218 Norwegian Caucasian children with JRA as compared with 194 Dutch Caucasian control individuals. Our observations are in agreement with the only previous study of MBL conducted in JIA patients [27]. In that study no association between *MBL2 *codon 54 mutations and JIA was found. We have now shown that JRA is also not associated with any of the other five known *MBL2 *SNPs.

The frequency of these mutations also did not differ from the frequencies identified in previously published Danish Caucasian control populations [10,11]. Over the past few years studies have been published that consistently reported similar frequencies in Caucasian populations of different countries [10,11,34]. Therefore, we assume that the frequencies of *MBL2 *gene polymorphism in the Caucasian Norwegian population do not differ from those in other Caucasian populations. Therefore, our present observations suggest that genetically determined MBL deficiency is not associated with increased susceptibility to JRA. Based on the number of included patients and control individuals, this study has 80% power when an odds ratio of 1.71 or greater for MBL deficiency is found.

Interestingly, children in the low *MBL2 *genotype group developed polyarthritis at a younger age than did children in the medium or high genotype groups. Previously, Garred and coworkers [23] showed that *MBL2 *exon 1 variant allele carrier status was associated with early age at onset of RA, which is the adult counterpart of polyarthritis [26]. Garred and coworkers hypothesized that MBL may delay the onset of RA but that it does not prevent the disease. The mechanism by which MBL deficiency might promote inflammation in immune-mediated inflammatory diseases such as RA and JRA is as yet unknown. MBL deficiency might lead to a diminished innate immunity, and subsequent increased risk for infections, as was previously remonstrated [15,16]. These infections may trigger JRA, as has been hypothesized previously [26]. Another possibility is that MBL is involved in the recognition of an infectious agent in the pathophysiology of JRA. Low or absent MBL plasma concentration leads to decreased complement activation and ineffective clearance of the pathogen or pathogen-derived antigens. The prolonged presence of infectious agents in the host may enhance synovial inflammation because of the proinflammatory effects of bacterial DNA and bacterial cell wall fragments [35,36]. Anti-MBL autoantibodies may also play a role, because elevated levels of anti-MBL autoantibodies were found in the sera of RA patients [37]. It is unclear at present whether MBL deficiency is indeed involved in the pathogenesis of RA or JRA, because the data reported are variable.

Furthermore, MBL deficiency does not appear to play a role once polyarthritis has developed, because no associations were found between *MBL2 *genotype and the laboratory variables or the remaining disease severity related clinical variables, such as PGA, CHAQ score, number of actively involved or affected joints, and number of patients with uveitis or remission. Consistent with the previous report by Barton and coworkers [25] on RA and MBL polymorphisms, we did not find an association between erosive joint destruction and MBL polymorphisms in patients with JRA.

In the oligoarthritis group, patients in the low genotype group were in remission more often (81%) than were the children in the medium or high genotype group (54% to 56%). In this regard, lack of the protein MBL in serum appears to be associated with a milder disease course or decreased inflammation. The possible explanation for these findings might be that MBL has an immunomodulating effect. MBL is present in synovial fluid and can bind potential causative agents in JRA including micro-organisms, cellular debris, and agalactosyl IgG (IgG-G0) [38,39]. Binding of MBL to agalactosyl IgG immune complexes may result in local complement activation and subsequent increased inflammation and thus active disease, whereas this is absent in the presence of very low levels of MBL [40]. Recently, Troelsen and colleagues [41] found that high serum levels of MBL and agalactosyl IgG were risk factors for ischaemic heart disease in RA patients. Besides, RA patients had higher MBL levels than did their relatives, suggesting that high MBL may trigger RA [39]. Harmful effects of high MBL levels have been shown in other disease entities as well. For instance, MBL deposits in the glomeruli can cause histological damage of kidneys, and activation of the lectin pathway by MBL can induce vascular tissue damage in myocardial ischemia-reperfusion injury and diabetes [42-44]. On the other hand, MBL deficiency might be associated with defective clearance of immune complexes and apoptotic cells, as seen in individuals with C1q deficiency. Because MBL and C1q are molecules with similar characteristics this might explain why during active disease CRP levels were increased in children in the low compared with the medium and high genotype groups. Remission rates were not associated with *MBL2 *genotype in patients with polyarthritis, possibly because more joints were affected.

## Conclusion

MBL appears to play a dual role in JRA. Genetically determined MBL deficiency does not increase susceptibility to JRA, but MBL does appear to have an immunomodulating effect. On the one hand children with low levels of MBL develop polyarthritis at younger age. In the case of MBL deficiency, potential explanations for this younger age at onset are increased susceptibility to infections, as a potential trigger of polyarthritis, or ineffective clearance of infectious agents in the pathophysiology of JRA. On the other hand, the low *MBL2 *expressing genotypes appear to be beneficial once oligoarthritis has developed, because they are associated with increased frequency of remission. An explanation may be that the local MBL itself may lead to complement-mediated inflammation in the synovium, sustaining active disease. If we are to discover the possible contribution of MBL to JRA disease severity, then we must study molecular mechanisms such as the interaction of MBL with immune complexes, the presence of anti-MBL autoantibodies and the role of activation of the complement system.

## Abbreviations

ANA = antinuclear antibody; CHAQ = Childhood Health Assessment Questionnaire; CRP = C-reactive protein; IQR = interquartile range; MBL = mannose-binding lectin; JIA = juvenile idiopathic arthritis; JRA = juvenile rheumatoid arthritis; PGA = physician's global assessment; RA = rheumatoid arthritis; RF = rheumatoid factor; SNP = single nucleotide polymorphism.

## Competing interests

The authors declare that they have no competing interests.

## Authors' contributions

The study was designed by KD, TK, PT and AS. They were all involved in the management of the study and in supporting other contributors. BF, OF and AS collected the clinical data. NB conducted the laboratory investigations. FF analyzed the data statistically and interpreted the results. She completed the first draft, written by KD. Finally, each author contributed to the writing of the final manuscript. They all read and approved this version of the manuscript and take full responsibility for it.

## References

[B1] Burgos-Vargas R, Vazquez-Mellado J (1995). The early clinical recognition of juvenile-onset ankylosing spondylitis and its differentiation from juvenile rheumatoid arthritis. Arthritis Rheum.

[B2] Ploski R, Vinje O, Ronningen KS, Spurkland A, Sorskaar D, Vartdal F, Forre O (1993). HLA class II alleles and heterogeneity of juvenile rheumatoid arthritis. DRB1*0101 may define a novel subset of the disease. Arthritis Rheum.

[B3] Schaller JG (1983). Pauciarticular arthritis of childhood (pauciarticular juvenile rheumatoid arthritis). Ann Pediatr (Paris).

[B4] Wilder RL, Crofford LJ (1991). Do infectious agents cause rheumatoid arthritis?. Clin Orthop Relat Res.

[B5] Turner MW (1996). Mannose-binding lectin: the pluripotent molecule of the innate immune system. Immunol Today.

[B6] Neth O, Jack DL, Dodds AW, Holzel H, Klein NJ, Turner MW (2000). Mannose-binding lectin binds to a range of clinically relevant microorganisms and promotes complement deposition. Infect Immun.

[B7] Saifuddin M, Hart ML, Gewurz H, Zhang Y, Spear GT (2000). Interaction of mannose-binding lectin with primary isolates of human immunodeficiency virus type 1. J Gen Virol.

[B8] Lipscombe RJ, Sumiya M, Hill AV, Lau YL, Levinsky RJ, Summerfield JA, Turner MW (1992). High frequencies in African and non-African populations of independent mutations in the mannose binding protein gene. Hum Mol Genet.

[B9] Madsen HO, Garred P, Kurtzhals JA, Lamm LU, Ryder LP, Thiel S, Svejgaard A (1994). A new frequent allele is the missing link in the structural polymorphism of the human mannan-binding protein. Immunogenetics.

[B10] Brouwer N, Dolman KM, van Zwieten R, Nieuwenhuys E, Hart M, Aarden LA, Roos D, Kuijpers TW (2006). Mannan-binding lectin (MBL)-mediated opsonization is enhanced by the alternative pathway amplification loop. Mol Immunol.

[B11] Kronborg G, Weis N, Madsen HO, Pedersen SS, Wejse C, Nielsen H, Skinhoj P, Garred P (2002). Variant mannose-binding lectin alleles are not associated with susceptibility to or outcome of invasive pneumococcal infection in randomly included patients. J Infect Dis.

[B12] Madsen HO, Garred P, Thiel S, Kurtzhals JA, Lamm LU, Ryder LP, Svejgaard A (1995). Interplay between promoter and structural gene variants control basal serum level of mannan-binding protein. J Immunol.

[B13] Madsen HO, Satz ML, Hogh B, Svejgaard A, Garred P (1998). Different molecular events result in low protein levels of mannan-binding lectin in populations from southeast Africa and South America. J Immunol.

[B14] Frakking FN, van de Wetering MD, Brouwer N, Dolman KM, Geissler J, Lemkes B, Caron HN, Kuijpers TW (2006). The role of mannose-binding lectin (MBL) in paediatric oncology patients with febrile neutropenia. Eur J Cancer.

[B15] Koch A, Melbye M, Sorensen P, Homoe P, Madsen HO, Molbak K, Hansen CH, Andersen LH, Hahn GW, Garred P (2001). Acute respiratory tract infections and mannose-binding lectin insufficiency during early childhood. JAMA.

[B16] Summerfield JA, Sumiya M, Levin M, Turner MW (1997). Association of mutations in mannose binding protein gene with childhood infection in consecutive hospital series. BMJ.

[B17] Garred P, Larsen F, Madsen HO, Koch C (2003). Mannose-binding lectin deficiency – revisited. Mol Immunol.

[B18] Lee YH, Witte T, Momot T, Schmidt RE, Kaufman KM, Harley JB, Sestak AL (2005). The mannose-binding lectin gene polymorphisms and systemic lupus erythematosus: two case-control studies and a meta-analysis. Arthritis Rheum.

[B19] Graudal NA, Homann C, Madsen HO, Svejgaard A, Jurik AG, Graudal HK, Garred P (1998). Mannan binding lectin in rheumatoid arthritis. A longitudinal study. J Rheumatol.

[B20] Graudal NA, Madsen HO, Tarp U, Svejgaard A, Jurik G, Graudal HK, Garred P (2000). The association of variant mannose-binding lectin genotypes with radiographic outcome in rheumatoid arthritis. Arthritis Rheum.

[B21] Jacobsen S, Madsen HO, Klarlund M, Jensen T, Skjodt H, Jensen KE, Svejgaard A, Garred P (2001). The influence of mannose binding lectin polymorphisms on disease outcome in early polyarthritis. TIRA Group. J Rheumatol.

[B22] Saevarsdottir S, Vikingsdottir T, Vikingsson A, Manfredsdottir V, Geirsson AJ, Valdimarsson H (2001). Low mannose binding lectin predicts poor prognosis in patients with early rheumatoid arthritis. A prospective study. J Rheumatol.

[B23] Garred P, Madsen HO, Marquart H, Hansen TM, Sørensen SF, Petersen J, Volck B, Svejgaard A, Graudal NA, Rudd PM, Dwek RA, Sim RB, Andersen V (2000). Two edged role of mannose binding lectin in rheumatoid arthritis: a cross sectional study. J Rheumatol.

[B24] Stanworth SJ, Donn RP, Hassall A, Dawes P, Ollier W, Snowden N (1998). Absence of an association between mannose-binding lectin polymorphism and rheumatoid arthritis. Br J Rheumatol.

[B25] Barton A, Platt H, Salway F, Symmons D, Lunt M, Worthington J, Silman A (2004). Polymorphisms in the mannose binding lectin (MBL) gene are not associated with radiographic erosions in rheumatoid or inflammatory polyarthritis. J Rheumatol.

[B26] Ravelli A, Martini A (2007). Juvenile idiopathic arthritis. Lancet.

[B27] Kang M, Wang HW, Cheng PX, Yin ZD, Li XO, Shi H, Hu XF (2006). Lack of association between mannose-binding lectin gene polymorphisms and juvenile idiopathic arthritis in a Han population from the Hubei province of China. Arthritis Res Ther.

[B28] Flato B, Smerdel A, Johnston V, Lien G, Dale K, Vinje O, Egeland T, Sorskaar D, Forre O (2002). The influence of patient characteristics, disease variables, and HLA alleles on the development of radiographically evident sacroiliitis in juvenile idiopathic arthritis. Arthritis Rheum.

[B29] Flato B, Lien G, Smerdel A, Vinje O, Dale K, Johnston V, Sorskaar D, Moum T, Ploski R, Forre O (2003). Prognostic factors in juvenile rheumatoid arthritis: a case-control study revealing early predictors and outcome after 14.9 years. J Rheumatol.

[B30] Brewer EJ, Bass J, Baum J, Cassidy JT, Fink C, Jacobs J, Hanson V, Levinson JE, Schaller J, Stillman JS (1977). Current proposed revision of JRA Criteria. JRA Criteria Subcommittee of the Diagnostic and Therapeutic Criteria Committee of the American Rheumatism Section of The Arthritis Foundation. Arthritis Rheum.

[B31] Singh G, Athreya BH, Fries JF, Goldsmith DP (1994). Measurement of health status in children with juvenile rheumatoid arthritis. Arthritis Rheum.

[B32] Tacx AN, Groeneveld AB, Hart MH, Aarden LA, Hack CE (2003). Mannan binding lectin in febrile adults: no correlation with microbial infection and complement activation. J Clin Pathol.

[B33] Bernig T, Breunis W, Brouwer N, Hutchinson A, Welch R, Roos D, Kuijpers T, Chanock S (2005). An analysis of genetic variation across the MBL2 locus in Dutch Caucasians indicates that 3' haplotypes could modify circulating levels of mannose-binding lectin. Hum Genet.

[B34] Garcia-Laorden MI, Pena MJ, Caminero JA, Garcia-Saavedra A, Campos-Herrero MI, Caballero A, Rodriguez-Gallego C (2006). Influence of mannose-binding lectin on HIV infection and tuberculosis in a Western-European population. Mol Immunol.

[B35] Schrijver IA, Melief MJ, Tak PP, Hazenberg MP, Laman JD (2000). Antigen-presenting cells containing bacterial peptidoglycan in synovial tissues of rheumatoid arthritis patients coexpress costimulatory molecules and cytokines. Arthritis Rheum.

[B36] van der Heijden I, Wilbrink B, Tchetverikov I, Schrijver IA, Schouls LM, Hazenberg MP, Breedveld FC, Tak PP (2000). Presence of bacterial DNA and bacterial peptidoglycans in joints of patients with rheumatoid arthritis and other arthritides. Arthritis Rheum.

[B37] Gupta B, Raghav SK, Agrawal C, Chaturvedi VP, Das RH, Das HR (2006). Anti-MBL autoantibodies in patients with rheumatoid arthritis: prevalence and clinical significance. J Autoimmun.

[B38] Saevarsdottir S, Vikingsdottir T, Valdimarsson H (2004). The potential role of mannan-binding lectin in the clearance of self-components including immune complexes. Scand J Immunol.

[B39] Saevarsdottir S, Steinsson K, Grondal G, Valdimarsson H (2007). Patients with rheumatoid arthritis have higher levels of mannan-binding lectin than their first-degree relatives and unrelated controls. J Rheumatol.

[B40] Malhotra R, Wormald MR, Rudd PM, Fischer PB, Dwek RA, Sim RB (1995). Glycosylation changes of IgG associated with rheumatoid arthritis can activate complement via the mannose-binding protein. Nat Med.

[B41] Troelsen LN, Garred P, Madsen HO, Jacobsen S (2007). Genetically determined high serum levels of mannose-binding lectin and agalactosyl IgG are associated with ischemic heart disease in rheumatoid arthritis. Arthritis Rheum.

[B42] Roos A, Bouwman LH, Munoz J, Zuiverloon T, Faber-Krol MC, Fallaux-van den Houten FC, Klar-Mohamad N, Hack CE, Tilanus MG, Daha MR (2003). Functional characterization of the lectin pathway of complement in human serum. Mol Immunol.

[B43] Hansen TK, Tarnow L, Thiel S, Steffensen R, Stehouwer CD, Schalkwijk CG, Parving HH, Flyvbjerg A (2004). Association between mannose-binding lectin and vascular complications in type 1 diabetes. Diabetes.

[B44] Jordan JE, Montalto MC, Stahl GL (2001). Inhibition of mannose-binding lectin reduces postischemic myocardial reperfusion injury. Circulation.

